# Adaptive Modelling of Mutated FMO3 Enzyme Could Unveil Unexplored Scenarios Linking Variant Haplotypes to TMAU Phenotypes

**DOI:** 10.3390/molecules26227045

**Published:** 2021-11-22

**Authors:** Simona Alibrandi, Fabiana Nicita, Luigi Donato, Concetta Scimone, Carmela Rinaldi, Rosalia D’Angelo, Antonina Sidoti

**Affiliations:** 1 Department of Biomedical and Dental Sciences and Morphofunctional Imaging, Division of Medical Biotechnologies and Preventive Medicine, University of Messina, 98125 Messina, Italy; salibrandi@unime.it (S.A.); fabiana.nicita@unime.it (F.N.); cscimone@unime.it (C.S.); crinaldi@unime.it (C.R.); rdangelo@unime.it (R.D.); asidoti@unime.it (A.S.); 2 Department of Chemical, Biological, Pharmaceutical and Environmental Sciences, University of Messina, 98125 Messina, Italy; 3 Department of Biomolecular Strategies, Genetics, Cutting-Edge Therapies, I.E.ME.S.T., 90139 Palermo, Italy

**Keywords:** *FMO3*, TMAU, in silico, proteomics, genetic variants

## Abstract

Background: Trimethylaminuria (TMAU) is a rare genetic disease characterized by the accumulation of trimethylamine (TMA) and its subsequent excretion trough main body fluids, determining the characteristic fish odour in affected patients. We realized an experimental study to investigate the role of several coding variants in the causative gene *FMO3*, that were only considered as polymorphic or benign, even if the available literature on them did not functionally explain their ineffectiveness on the encoded enzyme. Methods: Mutational analysis of 26 TMAU patients was realized by Sanger sequencing. Detected variants were, subsequently, deeply statistically and in silico characterized to determine their possible effects on the enzyme activity. To achieve this goal, a docking prediction for TMA/FMO3 and an unbinding pathway study were performed. Finally, a TMAO/TMA urine quantification by 1H-NMR spectroscopy was performed to support modelling results. Results: The *FMO3* screening of all patients highlighted the presence of 17 variants distributed in 26 different haplotypes. Both non-sense and missense considered variants might impair the enzymatic kinetics of *FMO3*, probably reducing the interaction time between the protein catalytic site and TMA, or losing the wild-type binding site. Conclusions: Even if further functional assays will confirm our predictive results, considering the possible role of *FMO3* variants with still uncertain effects, might be a relevant step towards the detection of novel scenarios in TMAU etiopathogenesis.

## 1. Introduction

Trimethylaminuria (TMAU) is a rare genetic disease characterized by the accumulation of trimethylamine (TMA) and its subsequent excretion trough breath sweat, saliva, vaginal secretions, and urine, determining a characteristic fish odour in affected patients [1]. TMA is a volatile tertiary amine, synthesized by gut microbiota. Choline, betaine, lecithin, and L-carnitine introduced by diet are TMA main precursors [2]. In a non-pathological condition, TMA is oxidized in an in-odorous molecule, the trimethylamine n-oxide (TMAO), by a hepatic microsomal enzyme known as Flavin-containing monooxygenase 3 (*FMO3*) [3].

TMA accumulation can be caused by both genetic and environmental factors, leading to TMAU different forms. To date, the primary form (TMAU1) is known to be determined by the *FMO3* enzyme deficiency or functional impairment, as consequence of the *FMO3* gene mutation induced effects [4]. The secondary form (TMAU2) is mainly caused by a gut dysbiosis condition. A higher relative abundance of some bacterial families could determine a TMA overproduction. Lastly, the transient form could be induced by excessive sweating due to intense physical effort, stress, mood disturbances, or influence of steroid hormones during the peri-menstrual period [5].

The absence of physical anomalies has brought this syndrome little medical interest, underestimated in its most purely psycho-affective aspects. Although this metabolic disorder is not physically disabling, it has a strong impact on the patient’s socio-psychic sphere. Social isolation, behaviour disorders, and depression are the main psychic manifestations and, in the most severe cases, suicide can also occur [6]. TMAU is particularly severe in children and adolescents due to the negative impact within the school context associated with humiliation experiences [7]. At this age, such social difficulties, in addition to the bad smell, lead to the consultation of a medical specialist. Unfortunately, there is no definitive therapy to date, only a combination of different approaches to reduce, temporarily, the bad smell [8]. Products such as chlorophyllin and activated charcoal are used to sequester the trimethylamine produced in the gut, riboflavin supplements are given to enhance residual *FMO3* enzyme activity, while antibiotics (metronidazole, amoxicillin, and neomycin) are used to reduce the levels of TMA-producing gut bacteria [9,10]. In addition, it is advisable to avoid foods such as shellfish, eggs, meat, legumes, and mushrooms, which contain TMA precursors (choline, carnitine, and betaine) [11].

Recently, a strong interest has arisen on the study of the secondary form of trimethylaminuria (TMAU2), but still little is known about the primary form (TMAU1), especially about the exact physiological and pathophysiological activity of the *FMO3* [12].

The *FMO3* enzyme catalyses the NADPH-dependent oxidative metabolism of a wide range of exogenous chemicals, including drugs, dietary-derived compounds, and environmental pollutants [13]. As already cited, it is responsible for the conversion of TMA, synthesized from precursors introduced with diet, in its oxidized and odourless form TMAO. Genetic variants in the *FMO3* gene, inherited in an autosomal recessive manner, could cause an enzymatic activity deficit and, consequently, a TMA accumulation [14,15,16].

To date, 91 *FMO3* variants were identified, and more than half of them are responsible for the TMAU phenotype (http://www.hgmd.cf.ac.uk/ac/index.php, accessed on 19 October 2021). Among the remaining variants, some are of uncertain clinical significance, while others are classified as “benign” and, therefore, should not affect the *FMO3* enzyme activity [17,18,19].

However, as such variants have a high frequency in TMAU patients, frequently without the contemporary presence of causative mutations, we hypothesized that their haplotypes could play a significant role in *FMO3* activity reduction or alteration. To confirm this hypothesis, we performed a proteomic in silico analysis, using different platforms and software, with the final aim of revealing how these variant combinations could influence the enzyme folding, also simulating its dynamic behaviour with the TMA substrate.

## 2. Results

### 2.1. Genotyping of TMAU Patients Revealed Different Haplotypes Made of Causative or Associated Variants Carried by FMO3

We screened the whole cohort of patients for the *FMO3* gene, founding 17 variants distributed in 26 different haplotypes. Among them, 11 haplotypes were made of missense or non-sense variants only (2, 4, 5, 6, 7, 8, 9, 10, 11, 12, and 13). Four patients (12, 17, 20, and 21) showed variants in homozygous condition, with a missense common to three patients (c. G472A p.E158K) and a splicing one common to two patients (c. 627 + 10 C > G). All known TMAU causative mutations were detected in heterozygous condition only. In detail, such variants were carried by patients 2 and 5 (c. 458C > T p.P153L), 9 (c. 1474C > T p.R492W), 10 (c. 713G > A p.R238Q and c. 713G > C p.R238P), 11 (c. 1424G > A G475D) and 13 (c. 1424G > A G475D and c. 713G > C p.R238P). The most complex haplotypes were exhibited by patients 5 and 8, which carried four common variants (c. G472A p.E158K, c. 485-22G > A, c. 1184-32_1184-31insT, and c. 923A > G p.E308G) and, respectively, c. 458C > T (p.P153L) and c. 441C > T (p.S147=). Finally, the most frequent variants resulted the c. G472A (p.E158K), carried by 18 TMAU patients. Further details are available in Table 1.

### 2.2. Primary Structural and Biochemical Analyses Highlighted Possible Altered Chemical-Physical Features in Mutated FMO3

The physicochemical properties of each mutated *FMO3* protein computed by ProtParam are shown in Table 2. The molecular weight ranged from 36,917 g/mol of Y331Stop to 60,090 g/mol of E158K_G475D. Theoretical isoelectric points (pI) for these proteins ranged from 6.26 of Y331Stop to 8.47 of E158K_E308G. The theoretical pI represents the pH at which a particular molecule or surface carries no net electrical charge, and it could help to understand the protein charge stability [20]. The mutated *FMO3* proteins of this study had GRAVY indexes ranging from −0.06 of D141V_G180V to −0.21 of Y331Stop. The GRAVY value for a peptide or protein is calculated as the sum of hydropathy values of all of the amino acids, divided by the number of residues in the sequence [21]. Thus, these low GRAVY ranges indicate the possibility of being a globular (hydrophilic) protein rather than membranous (hydrophobic). This information might be useful for localizing these proteins. The aliphatic index for all FMO3 proteins involved in this study ranged from 73.21 of Y331Stop to 83.16 of D141V_G180V. A high aliphatic index indicates that a protein is thermo-stable over a wide temperature range [22]. The aliphatic index of a protein is defined as the relative volume occupied by aliphatic side chains (alanine (Ala), valine (Val), isoleucine (Ile), and leucine (Leu)). It may be regarded as a positive factor for the increase in thermo-stability of globular proteins [22]. All proteins were stable (a protein whose instability index is <40 is predicted as stable [22]), with the instability index values for our hypothetical and conserved proteins range from 33.02 of Y331Stop to 35.12 of V267M. Further details on biochemical properties of wild-type and mutated *FMO3* proteins are available in Table S1.

### 2.3. Tertiary Structure Prediction of Mutated FMO3 Showed That Mutated Amino Acids Changed Their Distance from Surrounding Amino Acids

Due to the unavailability of *FMO3* 3D structure in the PDB Molecule Database, the i-TASSER servers were used to predict and model multiple domains of wild-type and mutated proteins with high confidence based on the high scoring template. Prediction by i-TASSER resulted the most confident, showing a normalized Z-score of 8.91 for wild-type structure and a mean value of 8.77 for the mutated ones.

**Figure 1:**
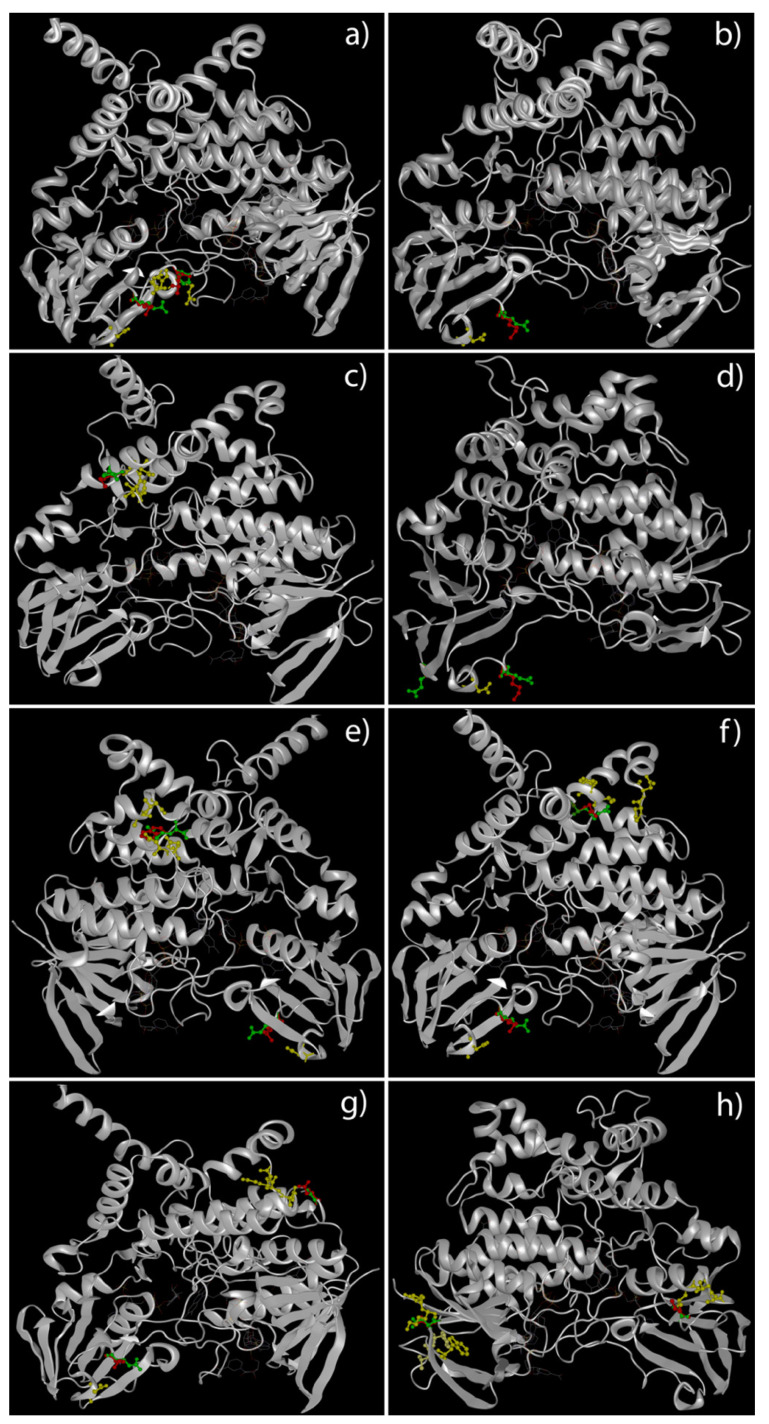
3D structural models of *FMO3* proteins in patients carrying missense variants. This panel highlights the predicted tertiary structure of *FMO3* in patients 5 (P153L and E158K) (**a**), 6 (E158K) (**b**), 7 (V257M) (**c**), 8 (E158K and E308G) (**d**), 9 (E158K and R492W (**e**), 10 (E158K and R238Q) (**f**), 11 (E158K and G475D) (**g**) and 12 (D141V and G180V) (**h**). Green ball-and-stick aa = wild-type aa. Red ball-and-stick aa = mutated aa. Yellow ball-and-stick aa = aa nearest to aa involved in variant.

**Figure 2:**
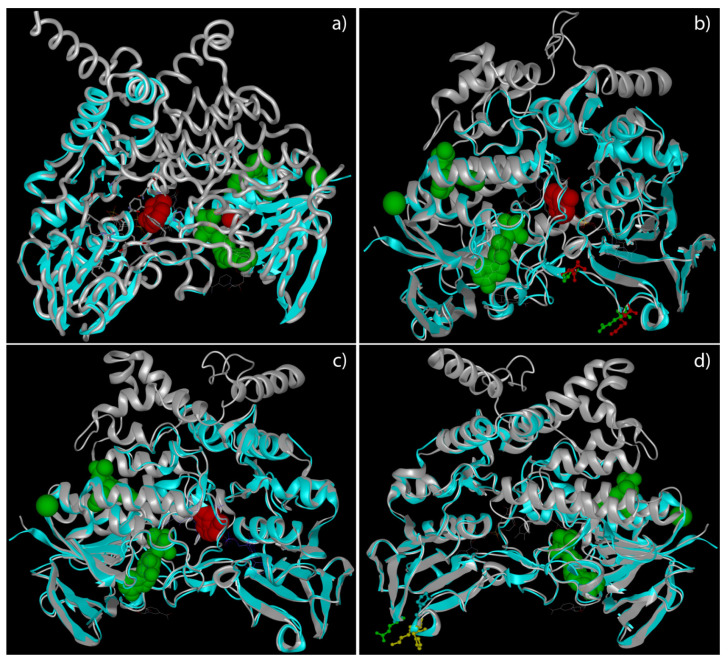
3D structural models of *FMO3* proteins in patients carrying non-sense variants. This panel highlights the predicted tertiary structure of *FMO3* in patients 1 (Y331Stop) (**a**), 2 (P153L, E158K and P380fs) (**b**), 3 (P380fs) (**c**), and 4 (E308G and P380fs) (**d**). The grey tube/ribbons represent the wild-type structures, while the light blue the over imposed mutated ones. The green spheres represent the predicted ligands/coenzymes/cofactors exclusive of wild-type *FMO3* (ADP, Na, and TMA), while the red spheres the predicted ones exclusive of different truncated FMO3 (Indole, Mg, and TMA; exceptions are represented by patient 3, who does not present the Mg, and by patient 4, who does not present any exclusive ligand compared to wild-type form of *FMO3*). Lateral chains of amino acids involved in missense variants are represented in green for the wild-type allele and in red for the mutated one. Yellow chains are characteristics of amino acids nearest to mutated ones.

A deep analysis of tertiary structures of *FMO3* mutated forms highlighted a global distribution of amino acids involved in missense variants along the outer domains of the enzyme (Figure 1). The truncated forms of *FMO3* determined by non-sense variants, instead, seem to lose the binding sites for co-enzymes and co-factors (Figure 2).

Looking inside the tridimensional structure and measuring the distance between the amino acid involved in missense variant and the surrounding ones, it was possible to evidence relevant changes in at least eight patients. Two altered forms of *FMO3* (E158K + P153L and E158K + G475D) showed a global reduction in distance between mutated amino acid and its nearest ones, while two others (E158K + R492W and E158K + R238Q) evidenced a wider distance between the same considered amino acids (Table 3).

### 2.4. Docking Analysis of FMO3 Forms Unveiled the Most Probable Binding Sites of TMA and How Considered Variants Could Impair This Binding

**Figure 3:**
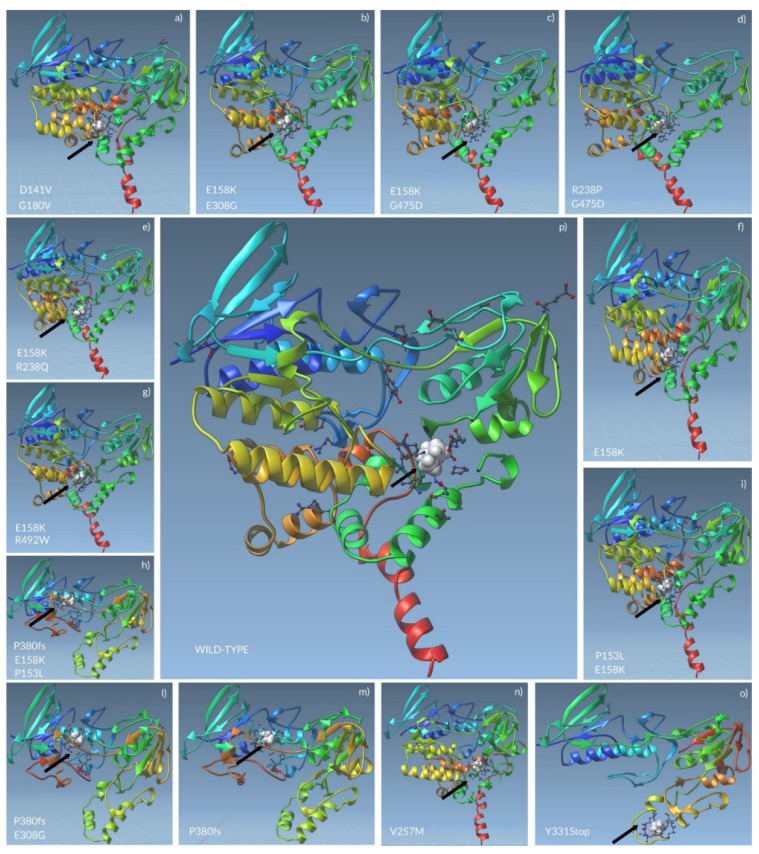
TMA docking to *FMO3* could involve different amino acids in mutated enzymes. The non-sense and missense variants carried by mutated *FMO3* (**a**–**o**) might shift the TMA binding sites far from the wild-type active site of the enzyme (**p**). The black arrows indicate the TMA (white spheres) bonded to the active site of *FMO3*, whose aa are represented as ball-and-stick. The other aa, represented as ball-and-stick, separated from the ones in the catalytic site, are the aa involved in mutations.

The docking analyses calculated a total of 10 modes with affinity (kcal/mol), Ki (µmol), and RMSD lower and upper bound (A) for each protein (Table S2). The mode presenting the lowest values for such variables, considered the most significant, was chosen for next steps (Table 4). The wild-type FMO3 highlighted that the TMA binding site could be made of Ser216, Gly217, Ser218, Trp219, Pro273, Asn275, Gly276, Leu278, Arg279, Lys280, Glu 281, and Pro 282. The same amino acids should constitute the active site of *FMO3* in patients 7 only (V257M). It is interesting to notice that patients 2, 10, 11, and 13 share the same aa in their *FMO3* active sites, as well as patient 8 with patient 9 and patient 4 with patient 5. On the contrary, little difference was found between active site aa of patient 8 and 9, which evidenced the loss of Lys422 if compared with patients 2, 10, 11, and 13. Moreover, patient 3 presented the loss of two aa (Gly38 and Val151) rather than patients 4 and 5. The absence of Arg417 altered the *FMO3* of patient 6 from the enzyme of patients 2, 10, 11, and 13. Finally, the only *FMO3* forms which presented unique aa in their active sites were the ones carried by patients 1 and 12. More details about aa of mutated *FMO3* active sites are available in Table 5 and Figure 3 and Figure S1.

### 2.5. The Unbinding Pathway Analyses of Mutated FMO3 Revealed an Impaired Interaction between TMA and Enzyme Active Site

The unbinding pathways emerged from Ligand Path Finder analyses showed the TMA could follow the identical route in patients 6, 8, 10, and 11, as well as in patients 9 and 13. Only patient 7 (carrying V257M) shares a pathway common to the wild-type *FMO3*.

In the wild-type *FMO3*, the TMA mainly interacted with the aa Ser216, Trp219, Leu278, and Lys 280. It went through the entire protein, also interacting with the Pro282 and the Glu 281, passing along the area containing the binding sites for FAD and NADP+.

The TMA unbinding pathway through the mutated *FMO3* of patients 6, 8, 10, and 11 was the exact opposite of the wild-type. Here, the TMA firstly interacted with the Trp419 (near to the FAD binding site), then bounced on the Arg279, the Asn245, and the Asn246.

A similar pathway was shown for patient 9, with the difference made of the interaction with the Leu278 after Trp419.

The *FMO3* carrying the V257M highlighted a TMA which interacted with Lys280, Pro 273, Asn275 and, at the end, with Ser216. During its exit path, the TMA seemed to be able to interact with NADP+. Even if such an exit way was opposite if compared to the wild-type, the TMA seemed to bind several aa of wild-type *FMO3* active site.

An interesting case is the one of patient 2, wherein the TMA interacted with Trp419 first, then with the Leu278 and the Arg279. After these interactions, due to the *FMO3* folding, Lys280 and Glu281 changed their conformation.

All the truncated forms of *FMO3* showed the loss of FAD binding sites.

In patient 1, the TMA interacted with Leu235 before exiting in a way similar to the wild-type (from top to bottom).

In patient 3, the TMA interacted with Ala377, Ala378, Ile379, Glu9, Ala10, Gly11, and, finally, with Glu281, before exiting as before.

The *FMO3* of patient 4 foresaw the interaction between TMA and Glu281 before Lys 280, preceding the exit in a way opposite to the wild-type *FMO3* (from the bottom to the top).

Finally, in patient 5, the TMA interacted with Ile87, Glu39, Gly38, Gly11, Ser85, and Asn84, tracing a route opposite to the wild-type *FMO3*, completely away from the enzyme active site.

**Figure 4:**
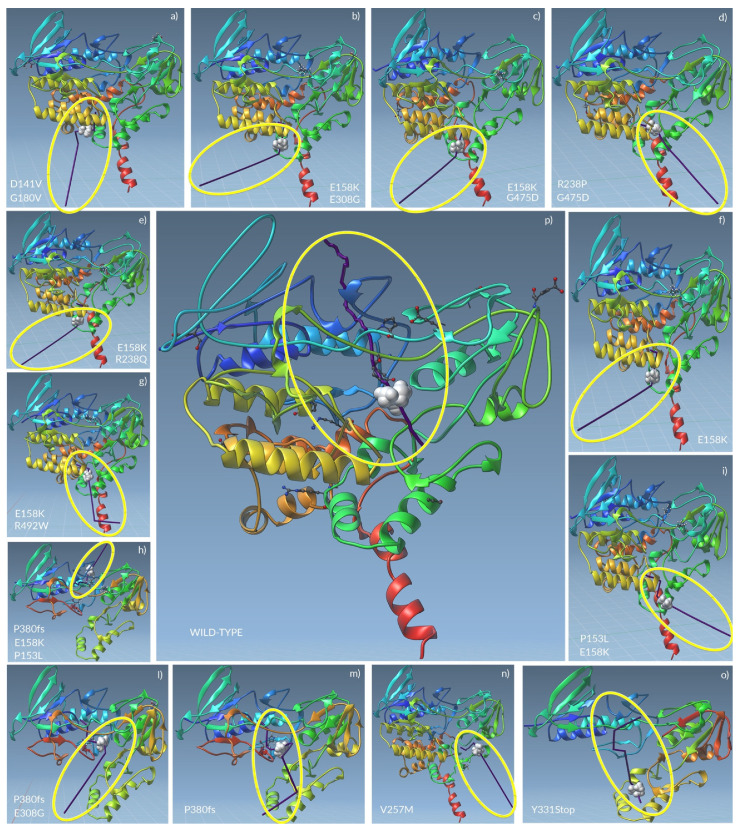
The unbinding pathways analyses showed that *FMO3* catalytic activity might not be fully performed. The TMA unbinding pathways through the whole *FMO3* mutated proteins (**a**–**o**) seemed to impair the enzyme functionality when compared to the wild-type one (**p**). The yellow circles indicate the unbinding pathway produced by pathlines (purple line) of TMA (white spheres) through the *FMO3*.

A graphical representation of unbinding pathways for wild-type and all mutated *FMO3* enzymes is shown in Figure 4, while detailed routes for all proteins are shown in Videos S1–S14.

### 2.6. NMR Spectra Functionally Confirmed an Altered Catalytic Activity of Mutated FMO3

**Figure 5:**
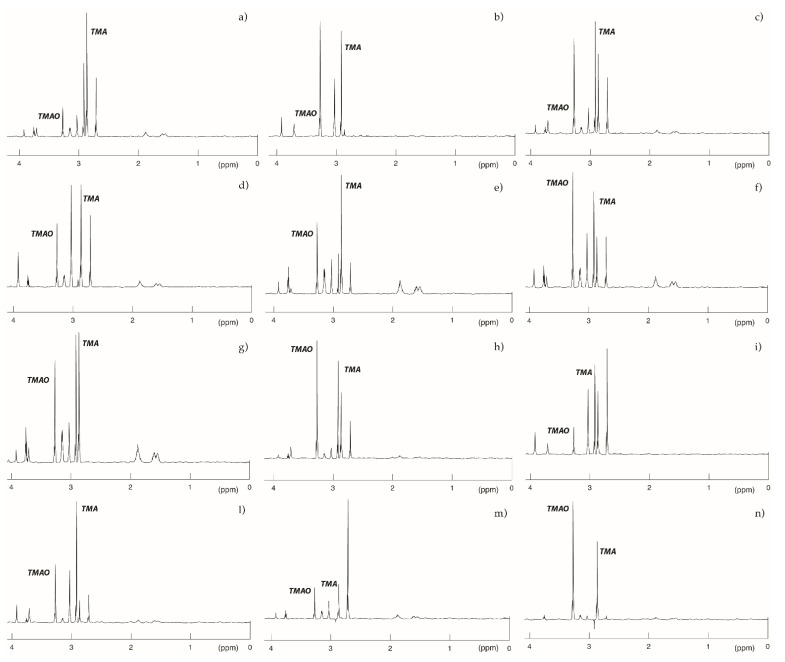
500 MHz ^1^H spectra of patients included in the study. Here, are represented the spectra resulting from ^1^H NMR spectroscopy of the urine collected from patients showing a unique *FMO3* variant haplotype. A healthy subject should present no peak for TMA and an intermediate peak for TMAO (not shown). Obtained results mostly confirmed genotyping and modelling analyses. (**a**) Patient 3; (**b**) Patient 10; (**c**) Patient 2; (**d**) Patient 9; (**e**) Patient 11; (**f**) Patient 6; (**g**) Patient 1; (**h**) Patient 8; (**i**) Patient 2; (**l**) Patient 4; (**m**) Patient 12; and (**n**) Patient 7. Metabolites peaks are assigned as follows: Trimethylamine-N-oxide (TMAO): 3.27 ppm; Creatinine (Cr): 3.06 ppm; Creatine (Cn): 3.04 ppm; Trimethylamine (TMA): 2.92 ppm; Dimethylamine (DMA): 2.73 ppm; and Citrulline (cit): 1.62 and 1.92 ppm.

The ^1^H-NMR spectra of urine samples from TMAU patients with unique haplotype mostly reflected what we already predicted with genotyping and modelling analyses (Figure 5).

The TMA peak at 2.92 ppm was much more intense in the urine samples from patients 1 (Figure 5a), 2 (Figure 5i), and 4 (Figure 5l), whose *FMO3* genotype was characterized by nonsense variants. Conversely, patients 6 (Figure 5f), 7 (Figure 5n), 8 (Figure 5h), and 10 (Figure 5b) showed a higher peak for TMAO, suggesting that *FMO3* variants carried by these subjects might only slightly alter the catalytic activity of the enzyme.

## 3. Discussion

The primary form of TMAU represent the genetic form of this social-impacting disease, and the one characterized by the most certain diagnosis. The latter consists of the screening of the *FMO3* gene for causative mutations [23]. However, despite this, many patients suffering of TMAU symptomatology did not carry causative mutations in the flavin containing monooxygenase 3 encoding gene. One hypothesis developed to explain such phenomenon relies on the possibility that other genes could be involved in TMAU etiopathogenesis. In the meantime, the role of many polymorphic variants able to impact on *FMO3* folding and activity is still unexplored, as well as the involvement of haplotypes in the onset and progression of trimethylaminuria [24].

It has been widely acknowledged that haplotype analysis in association studies can provide much more useful information than the information derived from single polymorphisms analysis [25]. The primary reason for considering the haplotype organization of variation resides in the fact that the folding kinetics, stability, and other physical features of a protein may depend on interactions between pairs or higher-order combination of aminoacidic sites; if such interactions are relevant, then haplotypes are of direct biological importance.

Thus, in our study we investigated 26 patients affected by TMAU, identifying 17 coding-sequence variants making 26 different haplotypes. Such variants were predicted to determine relevant conformational and biochemical changes in *FMO3* mutated structures, leading to possible impairment of its activity. By docking and unbinding analyses, we shed light on possible alterations involving the enzymatic role of *FMO3* on TMA.

Thanks to AutoDock Vina it was possible to determine the *FMO3* active site aminoacidic residues, so far unknown. Today, indeed, only isolated domains were homologically modelled on the basis of the bacterial *FMO3* [26].

The unbinding pathway analysis showed that TMA mainly interacts with Ser216, Gly217, Ser218, Trp219, Val220, Pro273, Asn 275, Gly 276, Leu278, Lys280, Glu281, and Pro282 amino acids of *FMO3*, suggesting that these aa might play an important role in the TMA oxidation process.

The single variant and haplotypic analyses results of the mutated *FMO3* revealed that V257M was the only one that does not appear to cause a change of the enzyme active site. Furthermore, from the ligand pathfinder analyses, it emerged that the TMA unbinding path routed in the *FMO3* carrying this variant, presented an opposite trajectory compared to that in wild-type enzyme. Probably, this variant might induce a protein folding change obstructing the TMA transit into the enzymatic pocket. Nevertheless, TMA seems to interact with most of the active site amino acids.

The situation resulted completely different for *FMO3* carrying Y331stop and P380Fs variants in haplotypes (P380Fs + E308G and P380Fs + P153L + E158K, respectively). It is already known from the literature that both variants lead to a truncated protein whose enzymatic activity is reduced or totally absent, depending on whether this variant is in a heterozygous or a homozygous condition [27]. However, the molecular mechanisms underlying this deficit are not yet fully known. As shown in Figure 2, the stop variants resulted in the loss of the binding site for the FAD prosthetic group, therefore blocking the TMA oxidation process.

Comparing results obtained from both docking and unbinding pathway analyses, it was clear that both the single E158K and this variant in combination with P153L, R238Q, G475D, E308G, and R492W, as well as the R238P + G475D haplotype might influence protein folding, causing a lower TMA-active site binding stability. Such hypothesis is supported by the fact that the TMA would seem to form more stable bonds with different amino acid residues than those present in the active site.

Furthermore, in all haplotypes, except for the D141V + G180V, Arg279 is the only active site amino acid with whom the TMA would appear to form a stable interaction.

All previously obtained results were mostly confirmed by ^1^H-NMR analysis, which underlined the role of nonsense variants, especially if associated with other missense ones, in the alteration of *FMO3* catalytic activity.

Thus, it seems to be clear that TMA might take a different path through the mutated FMO3 if compared to the wild-type enzyme. Both trajectory and direction are different.

Therefore, the described haplotypes might cause an enzymatic pocket narrowing, altering the TMA transit through the *FMO3*. Furthermore, such impairments could determine not only a different route for the TMA, but also a reduced interaction time of the ammine with the catalytic site of the enzyme. The final result of this altered enzymatic kinetics might be the reduced levels of TMAO and an accumulation of TMA, leading to the characteristic TMAU phenotype, whose different expression levels could depend on the particular haplotype carried by the patient.

## 4. Methods

### 4.1. Clinical Data

A cohort of 26 patients, with a mean age of 38 years and with a predominance of female sex, presented to our molecular genetic laboratories with a clinical diagnosis of suspected TMAU. All patients were subjected to a deep smell analysis, trying to classify the exact emitted odour, which is characteristic of TMAU phenotype. Then, several gastro-intestinal exams were performed, such as abdominal ultrasounds and microbiome analyses. Finally, a specific anamnestic study was realized in order to support the clinical diagnosis. Among personal details reported by patients, the intake of drugs and/or probiotics was carefully investigated, in order to rule out any confounding factors. A detailed list of patients’ clinical features is available in Table 6.

### 4.2. Mutational Analysis of FMO3 Gene

DNA was extracted from peripheral blood by using a QIAamp DNA Blood Midi Kit (Qiagen, Hilden, Germany) according to the manufacturer’s protocol. The *FMO3* (9 coding exons) gene was amplified using primers designed according to the genes published nucleotide sequence of GenBank (accessed on 18 July 2021). Sequences of such primers are available upon request.

PCR amplifications were carried out in a 50 µL solution containing 2 µL of each primer (10 µM), 0.8 µg of genomic, and 1.5 U MyTaq DNA Polymerase of (Bioline, London, UK). After an initial denaturation step at 95 °C for 1 min, the samples were subjected to 35 cycles of amplification consisting of 15 s of denaturation at 95 °C and 10 s of annealing. The annealing temperature was optimized for each primer set. Following PCR, 5 µL of amplified product was examined by electrophoresis on a 1% agarose gel. Direct sequencing was, then, performed using BigDye Terminator v.3.1 chemistry on a 3500 Genetic Analyzer (Thermo Fisher Scientific, Waltham, MA, USA).

Informed consent was obtained from all subjects involved in the study.

### 4.3. In Silico Structural Analyses of Wild-Type and Muted Forms of FMO3

In order to investigate the possible effects of found variants on the coding sequence and their impact on *FMO3* enzyme, a proteomic in silico approach was exploited.

ProtParam [28] permitted us to compare various physical and chemical parameters between the wild-type and the mutated *FMO3* protein sequences. The computed features included the molecular weight, theoretical pI, amino acid composition, atomic composition, instability index, aliphatic index, and grand average of hydropathicity (GRAVY).

The prediction of tertiary structure has been realized thanks to Iterative Threading ASSEmbly Refinement (i-TASSER) hierarchical approach [29]. 3D structures of wild-type and mutated *FMO3* were, then, analysed by UCSF ChimeraX [30], to visualize conformational changes and variations in intramolecular contacts.

### 4.4. Molecular Dynamics Analyses of FMO3/TMA Complex

The use of an unbinding pathway analysis tool is fundamental for the dynamical study of ligand-protein interaction, as the ligand binding might be simply treated as a two-state process between the unbound and bound states that form the stable basins of attraction. While the relative free energy difference between the two states controls the binding affinity, the kinetics of the binding is theoretically determined by the height of the relevant free energy barrier. Thus, the binding affinity can be exclusively governed by the endpoint states, while the binding kinetics is influenced by the detailed pathway connecting the endpoints.

In order to evaluate the possible docking between TMA and *FMO3* and the dynamical molecular modelling of their complex, the SAMSON-Connect platform for integrated molecular design (https://www.samson-connect.net, accessed on 19 October 2021), along with its extensions AutoDock Vina Extended [31], FIRE state updater [32], GROMACS Model Generator [33], and Ligand Path Finder [34] was exploited.

Before using the extensions described, each structural model was validated to find small free molecules and clashes (bound ligands which are superposed and covalently docked with each other and with the protein), check the bond length, and check for alternate locations which must be removed.

In detail, the AutoDock Vina Extended permitted a highly accurate docking between ligand and enzyme, reducing computation time with a multithreaded algorithm, and mixing a “physical” scoring with a machine learning one. The analyses were performed setting minimization to 5000 steps and docking exhaustiveness to 8. The analyses calculated a total of 10 modes with affinity (kcal/mol), Ki (µmol), and RMSD lower and upper bound (A).

The Ligand Path Finder permitted to validate the predicted complexes from the previous docking analysis and allowed the prediction of kinetics properties such as binding affinity, binding/unbinding rates, and binding potency. Moreover, the tool helped to identify protein sites which hinder/facilitate the ligand entry/exit or whether certain entry/exit routes are more favourable. Mode 1 was chosen for each protein–ligand complex to find unbinding paths with Ligand Path Finder. Before applying Ligand Path Finder, each protein model was minimized without changing its backbone conformation and without changing the ligand’s position. Next, another minimization was applied to refine the systems, using Universal Force Field (UFF) as the interaction model and FIRE as the state updater. Geometry optimization is a fundamental step in many molecular modelling applications, used to produce stable, realistic structures which correspond to energy minima, so FIRE (Fast Inertial Relaxation Engine) is an efficient optimizer for molecular structures that implements the FIRE minimizer described by Bitzek et al. [32]. In the FIRE properties, the following parameters have been set: step size (the initial step size given to the algorithm) = 1.00 fs, steps (frequency of the viewport update) = 100, and fixed (forces the time step to be constant).

After the minimization, the parameters were set in Ligand Path Finder as follows: use seed = 1234, ARAP-modelling iterations = 20, initial temperature (T) = 0.0010 K, temperature factor = 2.00, RRT extension step size = 1.0000 A, runs = 2, minimization iterations = 10, failures before T increases = 1, and max. ligand displacement = 40.00 A. The sampling region was set according to the region of interest for the analysis. A single atom in the ligand was set as active atoms (nitrogen) to control the movement of the ligand and extract it using the active atom from the receptor. The choice of fixed atoms may influence the resulting unbinding pathways and ensure that the protein does not drift along with the ligand, so a single atom in the centre of the protein from one of its backbones away from the binding site was set as the fixed one (CA from SER 195 Backbone). The maximum time per run was set to 4 h and 2 paths were found per model.

The Pathlines extension was used to create a visual model that represent the trajectory of the centre of mass of selected atoms along selected path and to understand the motion of atoms along a path.

### 4.5. Urine Sample Preparation and ^1^H NMR Spectroscopy

Once collected, the patients’ urine samples were immediately frozen at −20 °C and thawed at room temperature only before analysis. In detail, 600 μL aliquots were used directly for ^1^H-NMR analysis in 5 mm diameter NMR tubes, together with 100 μL of deuterium oxide 99.96% (Eurisotop) as an internal field-frequency lock. Dietary overload (a 3 day, amine-rich diet) was performed by evaluated patients. Fish, cabbage, eggs, and crustaceans were recommended. The urine sample was collected on the fourth day.

NMR experiments were performed in a Bruker AVANCE III spectrometer (Bruker Biospin) operating at 500 MHz, set with a 5 mm gradient indirect detection probe and a probe temperature of 300 K. The one-dimensional proton spectra were acquired with 64 scans, 32 K data points, and a spectral width of 5000 Hz. A conventional proton 90° pulse with a relaxation delay of 2 s was applied. The water signal was suppressed by irradiation at the water resonance frequency (i.e., with a presaturation sequence). Resonances were assigned by reference to a spectral database of standard chemical shifts [35]. The Cr resonance at 3.05 ppm was used as an internal chemical shift reference. In these conditions, TMA resonance was detected at 2.92 ± 0.02 ppm, while TMAO was detected at approximately 3.27 ± 0.03 ppm, depending on the urine’s pH. The metabolite peaks for Cr, TMA, and TMAO were quantified by integration. The total amounts of TMA and TMAO excreted were normalized against the amount of Cr eliminated, in order to estimate the quantity considering the glomerular filtration. The values considered for healthy subjects were TMA/Cr < 10, TMAO/Cr ranging from 50 to 1000, and TMA/TMAO < 0.1 [36].

## 5. Conclusions

We realized an experimental study to investigate the role of several coding variants carried by the *FMO3* gene in a cohort of patients affected by trimethylaminuria. Evaluated variants were only considered as polymorphic or benign, but the available literature on them has never been able to functionally explain their ineffectiveness on the encoded enzyme.

Both molecular modelling and ^1^H-NMR spectroscopy suggested that both non-sense and missense considered variants might impair the enzymatic kinetics of *FMO3* with different severities, probably reducing the interaction time between the protein catalytic site and TMA, or losing the wild-type binding site.

However, we cannot assert with certainty that the same effect, in vivo, is limited to their presence, as there will be other factors involved into *FMO3* catalysis alterations, e.g., variant impact on co-enzymes and co-factors binding sites and activity.

Despite this, considering the possible role of *FMO3* variants with still uncertain effects, might be a relevant step towards the detection of novel scenarios in TMAU etiopathogenesis.

## Figures and Tables

**Table 1:** *FMO3* detected variants genotyping and distribution through the cohort of patients. Seventeen variants were identified during *FMO3* genotyping of all twenty-six considered patients. DM = disease mutation (HGMD). DM? = disease mutation without certain evidence (HGMD). DFP = disease-associated polymorphism with additional supporting functional evidence (HGMD). DP = disease-associated polymorphism (HGMD). FP = in vitro/laboratory or in vivo functional polymorphism (HGMD). Variants without HGMD acronym are not yet fully evaluated. HET = heterozygous variant. HOM = homozygous variant.

**ID**	***FMO3* Variants**																
	c. G472A (p.E158K) (DFP)	c.627+10 C>G (DM?)	c.485-22G>A	c.1184-32_1184-31insT	c.923A>G (p.E308G) (DFP)	c.769G>A (p.V257M) (FP)	c.458C>T (p.P153L) (DM)	c.441C>T (p.S147=) (DP)	c.993_994delTA (p.Tyr331Stop)	c.1474C>Tp.R492W (DM)	c.1139_1140del (p.Pro380fs)	c.713G>A (p.R238Q) (DM)	c.1424G>A (G475D) (DM)	c.713G>C (p.R238P) (DM)	c.855C>T (Asn285=)	c.422A>T (Asp141Val)	c.539G>T (Gly180Val)
1									HET								
2	HET						HET				HET						
3											HET						
4					HET						HET						
5	HET		HET	HET	HET		HET										
6	HET	HET															
7		HET	HET			HET											
8	HET		HET	HET	HET			HET									
9	HET		HET							HET							
10	HET											HET		HET			
11	HET												HET				
12								HOM							HOM	HOM	HOM
13													HET	HET			
14	HET	HET			HET												
15	HET	HET				HET											
16	HET				HET												
17	HOM	HOM	HOM														
18	HET	HET				HET											
19	HET	HET															
20	HOM	HOM															
21	HOM	HET						HET									
22	HET		HET		HET												
23						HET											
24						HET											
25	HET		HET														
26	HET	HET	HET	HET													

**Table 2:** Biochemical and physical changes prediction between wild-type and mutated *FMO3*. The ProtParam computed features included the molecular weight, theoretical pI, amino acid composition, atomic composition, instability index, aliphatic index, and grand average of hydropathicity (GRAVY). Analyses of such biochemical and physical parameters showed a decreased global instability from wild-type to mutated *FMO3* protein encoded by *FMO3* gene carrying SNVs only.

	WILD	P153L_E158K	V267M	E158K	E158K_E308G	E158K_R492W	E158K_R238Q	E158K_G475D	D141V_G180V	Y331Stop	R238P_G475D	P380fs_P153L_E158K	P380fs	P380fs_E308G
***Residues***	532	532	532	532	532	532	532	532	532	330	532	381	381	381
***MW* (g/mol)**	60,033	60,048	60,065	60,032	59,960	60,062	60,004	60,090	60,059	36,917	60,032	42,556	42,541	42,469
**1 μg *to* pmol**	16.6	16.65	16.65	16.66	16.68	16.65	16.67	16.64	16.65	27.09	16.66	23.5	23.51	23.55
***Net charge (pH =* 7*)***	2.17	4.17	2.17	4.17	5.17	3.17	3.17	3.17	3.17	−2.74	0.17	−0.65	−2.65	−1.65
***pI***	7.9	8.33	7.9	8.33	8.47	8.15	8.15	8.15	8.16	6.26	7.09	6.76	6.33	6.52
***Avg. hydropathy (GRAVY)***	−0.08	−0.07	−0.08	−0.08	−0.07	−0.07	−0.08	−0.09	−0.06	−0.21	−0.08	−0.10	−0.11	−0.10
***Aliphatic index***	82.07	82.8	81.52	82.07	82.07	82.07	82.07	82.07	83.16	73.21	82.07	80.05	79.03	79.03
***Instability index***	34.24	33.64	35.12	33.87	33.19	33.19	33.59	33.75	34.37	33.02	34.48	33.48	34.31	33.36
**1 mg/mL *to A₂₈₀***	1.46	1.46	1.46	1.46	1.46	1.55	1.46	1.46	1.46	1.23	1.46	1.14	1.14	1.14
***ε₂₈₀* (M⁻¹ cm⁻¹)**	87,485	87,485	87,485	87,485	87,485	92,85	87,485	87,485	87,485	45,420	87,485	48,400	48,400	48,400
**1 *A₂₈₀ to* mg/mL**	0.69	0.69	0.69	0.69	0.69	0.65	0.69	0.69	0.69	0.81	0.69	0.88	0.88	0.88
**1 mg/mL *to A₂₈₀ (red.)***	1.45	1.45	1.45	1.45	1.45	1.54	1.45	1.45	1.45	1.22	1.45	1.13	1.13	1.13
***ε₂₈₀* (M⁻¹ cm⁻¹) *(red.)***	86,860	86,860	86,860	86,860	86,860	92,360	86,860	86,860	86,860	44,920	86,860	47,900	47,900	47,900
***1 A₂₈₀ to* mg/mL *(red.)***	0.69	0.69	0.69	0.69	0.69	0.65	0.69	0.69	0.69	0.82	0.69	0.89	0.89	0.89
***Features***	NAP, FAD, WP5, OXY, ADP, IND, NA, 23 helices, 22 strands	NAP, FAD, WP5, OXY, ADP, IND, NA, 46 helices, 44 strands	NAP, FAD, WP5, OXY, ADP, IND, NA, 46 helices, 44 strands	NAP, FAD, WP5, OXY, ADP, IND, 46 helices, 44 strands	NAP, FAD, WP5, OXY, ADP, IND, NA, 46 helices, 44 strands	NAP, FAD, WP5, OXY, ADP, IND, NA, 46 helices, 44 strands	NAP, FAD, WP5, OXY, ADP, IND, NA, 46 helices, 44 strands	NAP, FAD, WP5, OXY, ADP, IND, NA, 46 helices, 44 strands	NAP, FAD, WP5, OXY, ADP, IND, NA, 46 helices, 44 strands	NAP, FAD, OXY, MG, IND, MMZ, CYH, 11 helices, 20 strands	NAP, FAD, WP5, OXY, ADP, IND, NA, 46 helices, 44 strands	NAP, FAD, WP5, OXY, ADP, IND, NA, 28 helices, 52 strands	NAP, FAD, OXY, IND, MMZ, CYH, 14 helices, 26 strands	NAP, FAD, WP5, OXY, ADP, IND, NA, 28 helices, 52 strands

**Table 3:** *FMO3* missense variants highlighting the highest distance differences between changed amino acid and nearest ones. The wild-type alleles compared to mutated ones showed possible interactions with difference nearest amino acids, also involving different atoms or functional groups (between brackets). Side chains functional groups are reported following the IUPAC-IUB Commission on Biochemical Nomenclature. Wt = wild-type amino acid and atom/functional group involved in a possible interaction with the nearest aa. Mut = mutated amino acid and atom/functional group involved in a possible interaction with the nearest aa.

ID	Variant	Wt	Nearest a.a.	a.a. Distance (Å)	Mut	Nearest a.a.	a.a. Distance (Å)
2	P153L	Pro153 (CG)	Arg174 (NE)	4.33	Leu153 (CD1)	Arg174 (NE)	5.20
		Pro153 ©	Leu155 (N)	3.20	Leu153 (CD2)	Leu155 (CD2)	1.71
		Pro153 (CB)	His172 (CE1)	3.32	Leu153 (CB)	His172 (CG)	4.00
	E158K	Glu158 (N)	Asn164 (OD1)	5.94	Lys158 (CA)	Asn164 (ND2)	5.71
4	E308G	Glu308	/	/	Gly308	/	/
5	P153L	Pro153 (CG)	Arg174 (NE)	4.33	Leu153 (CD1)	Arg174 (NE)	5.20
		Pro153 (C)	Leu155 (N)	3.20	Leu153 (CD2)	Leu155 (CD2)	1.71
		Pro153 (CB)	His172 (CE1)	3.32	Leu153 (CB)	His172 (CG)	4.00
	E158K	Glu158 (N)	Asn164 (OD1)	5.94	Lys158 (CA)	Asn164 (ND2)	5.71
6	E158K	Glu158 (N)	Asn164 (OD1)	5.94	Lys158 (CA)	Asn164 (ND2)	5.71
7	V257M	Val257 (N)	Tyr256 (C)	1.35	Met257 (N)	Tyr256 (C)	1.2
			Asp253 (O)	2.97		Asp253 (O)	3.13
		Val257 (CG2)	Val277 (N)	4.03	Met257 (CE)	Val277 (N)	4.09
8	E158K	Glu158 (N)	Asn164 (OD1)	5.94	Lys158 (CA)	Asn164 (ND2)	5.71
	E308G	Glu308	/	/	Gly308	/	/
9	E158K	Glu158 (N)	Asn164 (OD1)	5.94	Lys158 (CA)	Asn164 (ND2)	5.71
	R492W	Arg492 (CD)	Asp76 (OD2)	3.07	Tyr492 (NE1)	ASp76 (OD2)	2.53
		Arg492 (NH2)	Thr488 (OG1)	2.6	Trp492 (CH2)	Thr488 (O)	3.11
		Arg492 (NH1)	Ala485 (O)	2.72	Trp492 (N)	Ala485 (CB)	4.3
		Arg492 (NE)	Glu65 (OE2)	2.68	Trp492 (CE3)	Glu65 (OE2)	2.92
10	E158K	Glu158 (N)	Asn164 (OD1)	5.94	Lys158 (CA)	Asn164 (ND2)	5.71
	R238Q	Arg238 (NH2)	Pro445 (O)	4.95	Gln238 (OE1)	Pro445 (O)	7.59
			Asn446 (N)	7.15		Asn446 (N)	9.81
		Arg238 (CG)	Val261 (O)	3.12	Gln238 (CB)	Val461 (O)	3.59
		Arg238 (CB)	Tyr462 (O)	3.06		Tyr462 (O)	2.59
11	E158K	Glu158(N)	Asn164 (OD1)	5.94	Lys158 (CA)	Asn164 (ND2)	5.7
	G475D	Gly475 (N)	Pro445 (N)	7.07	Asp475 (OD2)	Pro445 (N)	5.90
		Gly475 (CA)	Lys444 (N)	6.18		Lys444 (N)	3.83
			Ala443 (CA)	4.30		Ala443 (CA)	3.02
			Gly442 (O)	4.52		Gly442 (O)	1.41
12	D141V	D141 (N)	Lys4 (O)	2.87	Val141 (N)	Lys4 (O)	3.02
			Val139 (C)	4.52		Val139 (C)	4.50
			Phe140 (C)	1.34		Phe140 (C)	1.25
		D141 (OD1)	Lys3 (CA)	3.52	Val141 (CG1)	Lys3 (CG)	3.88
		D141 (C)	Trp125 (CD1)	4.59	Val141 (C)	Trp125 (CD1)	4.44
	G180V	Gly180 (N)	Leu203 (CD2)	6.28	Val180 (N)	Leu203 (CD2)	6.22
			Ala207 (N)	8.42		Ala207 (N)	8.28
			Thr206 (OG1)	5.63		Thr206 (OG1)	5.49
13	R238P	Arg238 (NH1)	Ile447 (CD)	3.62	Pro238 (CB)	Ile447 (CD)	6.78
		Arg238 (NH2)	Pro445 (O)	4.95	Pro238 (CG)	Pro445 (O)	9.01
		Arg238 (N)	Gly464 (O)	3.44	Pro238 (CD)	Gly464 (O)	2.74
		Arg238 (N)	Cys466 (N)	4.56	Pro238 (CD)	Cys466(N)	3.65
	G475D	Gly475 (N)	Pro445 (N)	7.07	Asp475 (OD2)	Pro445 (N)	5.90
		Gly475 (CA)	Lys444 (N)	6.18		Lys444 (N)	3.83
			Ala443 (CA)	4.30		Ala443 (CA)	3.02
			Gly442 (O)	4.52		Gly442 (O)	1.41

**Table 4:** Best significant modes of TMA/*FMO3* docking models. AutoDock Vina permitted to compute 10 different modes for docking analysis between TMA and *FMO3*. This table shows the most reliable models, based on energetic variables. K_i_ = inhibition constant. RMSD values are calculated relative to the best mode (the first model) and use only movable heavy atoms (i.e., only ligand atoms, not hydrogen). RMSD upper bound matches each atom in one conformation with itself in the other conformation, ignoring any symmetry. RMSD lower bound matches each atom in one conformation with the closest atom of the same element type in the other conformation.

Protein	Affinity (kcal/mol)	K_i_ (µmol)	Lower Bound of the RMSD from This Ligand’s Best Mode (A)	Upper Bound of the RMSD from This Ligand’s Best Mode (A)
WILD-TYPE	−2.103	28,756.2	0.000	0.000
D141V_G180V	−2.160	26,096.6	0.000	0.000
E158K_E308G	−2.242	22,732.4	0.000	0.000
E158K_G475D	−2.239	22,844.0	0.000	0.000
E158K_R238Q	−2.240	22,789.5	0.000	0.000
E158K_R492W	−2.242	22,739.5	0.000	0.000
E158K	−2.240	22,791.2	0.000	0.000
P153L_E158K	−2.240	22,807.7	0.000	0.000
P380fs	−2.079	29,951.3	0.000	0.000
P380fs_P153L_E158K	−2.082	29,760.4	0.000	0.000
P380fs_E308G	−2.082	29,789.6	0.000	0.000
R238P_G475D	−2.242	22,736.2	0.000	0.000
V257M	−2.242	22,749.4	0.000	0.000
Y331Stop	−2.033	32,327.0	0.000	0.000

**Table 5:** Mutated *FMO3* showed different amino acids predicted for the TMA binding sites. Different haplotypes present in the mutated forms of *FMO3* were predicted to change the aa characteristic of wild-type enzyme catalytic site, probably leading to an impairment of *FMO3* activity. The “X” indicates that the specific aa in the related row is an integral part of the *FMO3* active site when the variant/s reported in the columns is/are carried by the *FMO3* gene.

	*FMO3* Haplotypes													
Active site a.a. Residues	Wild-Type	Y331Stop	P153L + E158K	P380Fs	P380Fs + E308G	P153L + E158K + P380Fs	E158K	V257M	E308G + E158K	R492W + E158K	R238Q + E158K	G475D + E158K	D141V + G180V	R238P + G475D
Gly 9				X	X	X								
Ala 10				X	X	X								
Gly 11				X	X	X								
Val 12				X	X	X								
Ser 13				X	X	X								
Gly 14				X	X	X								
Glu 32				X	X	X								
Gly 38					X	X								
Gly 39				X	X	X								
Leu 40				X	X	X								
Ala 52				X	X	X								
Cys 146				X	X	X								
Ser 147				X	X	X								
Gly 148				X	X	X								
Hys 149				X	X	X								
Val 151					X	X								
Ser 216	X							X						
Gly 217	X							X						
Ser 218	X							X						
Trp 219	X							X						
Gly 240		X												
Leu 243		X												
Lys 244		X												
Leu 247		X												
Ile 251		X												
Ser 252		X												
Asp 253		X												
Leu 255		X												
Tyr 256		X												
Gln 259		X												
Pro 273	X							X						
Asn 275	X							X						
Gly 276	X							X						
Leu 278	X							X						
Arg 279	X	X	X				X	X	X	X	X	X		X
Lys 280	X							X						
Glu 281	X							X						
Pro 282	X							X						
Leu 352													X	
Phe 353													X	
Lys 354													X	
Gly 355													X	
Phe 371													X	
Val 372													X	
Ser 381				X	X	X								
Lys 412			X				X		X	X	X	X	X	X
Met 413													X	
Lys 415			X				X		X	X	X	X		X
Lys 416			X				X		X	X	X	X	X	X
Arg 417			X						X	X	X	X		X
Trp 419			X				X		X	X	X	X		X
Phe 420			X				X		X	X	X	X		X
Lys 422			X				X				X	X		X
Thr 425			X				X		X	X	X	X		X
Ile 426													X	
Gln 427			X				X		X	X	X	X		X
Thr 428			X				X		X	X	X	X	X	X
Asp 429			X				X		X	X	X	X		X
Tyr 433													X	

**Table 6:** Clinical features of the 26 TMAU patients involved in this study. Signs, symptoms, and pathological features listed are those most frequently observed in TMAU affected individuals, as well as several ones specific of particular cases.

ID	Age	Smell Description	Intestinal Disorders	Liver Diseases	Age of Onset	Drugs	Supplements/Probiotics
1	9	Rotten fish	/	/	6 months	/	/
2	42	Rotten fish	Colitis	no	7/8 years	no	no
3	48	Genital fishy odour, body garbage odour, and scalp acid/sulphur odour	Irritable colon	no	10 years	Eutirox, Prisma 50	no
4		Rotten fish					
5	36	Rotten fish	Moderate degree of dysbiosis	no	2/3 years		
6	71	Rotten fish	Megacolon, anorectal stenosis, constipation, and congenital sacro-coccygeal malformation with fistulas	/	2/3 years	/	/
7	38	Rotten fish	/	/	37 years	Atazanavir, Abacavoir, and Lamivudin	Riboflavin
8	45	Rotten fish	/	/	38 years	Oral contraceptives	/
9	21	Rotten fish	no	no	6/7 years	no	no
10	73	Rotten fish	no	no	63 years	/	no
11	51	Fish	no	no	2/3 years	no	no
12	20	Rotten fish	no	no	6 months	no	no
13	28	Fish	no	no	After weaning	no	no
14	12	Rotten fish	/	/	5 years	/	/
15	31	Rotten fish, garbage, and garlic	Moderate dysbiosis	no	17 years	/	/
16	51	Rotten fish	/	/	44 years	Medicinal herbs	/
17	26	Rotten fish	no	no	14 years	Psychotropic drugs and tranquilizers	
18	48	Rotten fish, faecal odour	Mild dysbiosis, slow oro-caecal transit	no	/	/	
19	52	Rotten fish	Mild dysbiosis	no	6 years	/	Flebinic (carnitine-based supplement)
20	24	Rotten fish	no	no	After weaning	no	Activated carbon
21	38	Rotten fish	no	no	34 years	no	no
22	13	Rotten fish	no	no	10 years	no	no
23	28	Rotten fish	no	no	28 years	no	no
24	27	Rotten fish, faecal odour, rot, and sulphur	Mild dysbiosis	no	4 years	no	no
25	47	Rotten fish	/	/	39 years	/	/
26	71	Rotten fish	/	/	2/3 years	/	Assumption of a.a.

## Data Availability

All data generated or analysed during this study are included in this published article and its supplementary information files.

## Supplementary Materials

Figure S1: TMA docking to *FMO3* could involve different amino acids in mutated enzymes (details). Table S1: Secondary structure and biochemical comparisons between wild-type and mutated *FMO3* by different algorithms. Table S2: AutoDock Vina Extended docking analysis of wild-type and mutated *FMO3* proteins. Videos S1–S14: Unbinding pathways of wild-type and mutated *FMO3* enzymes.

## Sample Availability

Samples of the compounds are not available from the authors.
