# Supplementary material for: Adaptive Modelling of Mutated FMO3 Enzyme Could Unveil Unexplored Scenarios Linking Variant Haplotypes to TMAU Phenotypes
Source: Molecules. 2021 Nov 22;26(22):7045. doi: 10.3390/molecules26227045 (PMC8618768; doi:10.3390/molecules26227045)
Supplement: Supplementary file 1 [file molecules-26-07045-s001.zip › Supplementary Materials/Supplementary_Figures/Figure S1.pdf]

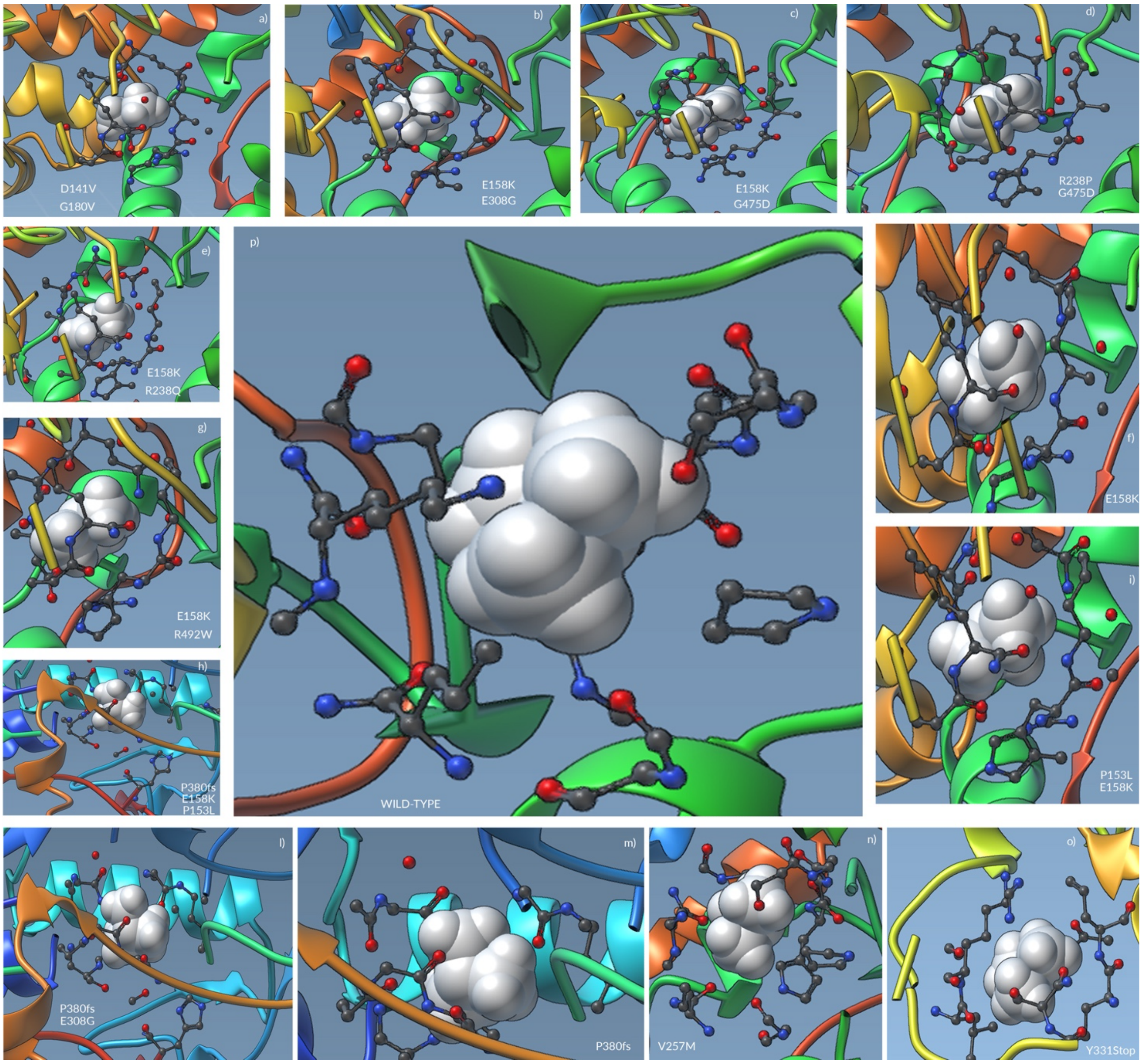

Figure S1. TMA docking to fmo3 could involve different amino acids in mutated enzymes (details). The non-sense and missense variants carried by mutated *FMO3* (a-o) might shift the TMA binding sites far from the wild-type active site of the enzyme (p). The ball-and-stick aa made relevant parts of the fmo3 TMA binding site. For more details about these aa refer to Table 5.
